# Deviations from recommended use of liposomal bupivacaine: a real-world pharmacovigilance study using the FAERS database

**DOI:** 10.3389/fmed.2026.1851787

**Published:** 2026-05-29

**Authors:** Wei Ma, Jing Lu, Cheng Shen, Haoshi Gao, Wei Gao

**Affiliations:** 1 School of Pharmacy, Guangzhou Xinhua University, Guangzhou, China; 2 Pharmacy Department, Liwan Central Hospital of Guangzhou, Guangzhou, China; 3 Department of Pharmacy, The Affiliated Children’s Hospital of Xiangya School of Medicine, Central South University (Hunan Children’s Hospital), Changsha, China; 4 School of Pharmacy, Guangdong Pharmaceutical University, Guangzhou, China; 5 Department of Urology, Liwan Central Hospital of Guangzhou, Guangzhou, China

**Keywords:** disproportionality analysis, FAERS database, liposomal bupivacaine, medication error, off-label use, pharmacovigilance

## Abstract

**Background:**

Liposomal bupivacaine (L-BUP) is a widely used long-acting local anesthetic, but real-world data on its post-marketing off-label use and medication error risks remain limited. This study aimed to characterize these risks to inform rational clinical use and post-marketing risk management of improved new drug formulations.

**Methods:**

L-BUP-related safety reports were extracted from the FAERS database (Q1 2004–Q2 2025). Four disproportionality analysis methods (ROR, PRR, BCPNN, and MGPS) were applied, with a positive signal defined as simultaneously meeting all four predefined criteria. Risk signals were mapped via Medical Dictionary for Regulatory Activities (MedDRA) Preferred Terms (PTs) and Standardized MedDRA Queries (SMQs). Conventional bupivacaine (C-BUP) was used as a comparator in the sensitivity analysis to verify the specificity of risk signals.

**Results:**

Among 19,252,329 included FAERS reports, 4,151 listed L-BUP as the primary suspect drug. Of the top 20 L-BUP positive signals, 35% were associated with deviations from recommended use. Notably, “Labeled drug–drug interaction medication error” exhibited the strongest signal (ROR = 332.7), while “Off-label use” had the highest reporting frequency, with 238 cases. At the SMQ level, medication errors yielded a positive safety signal (ROR = 4.17). Sensitivity analysis confirmed that C-BUP presented a negative signal for medication errors (ROR = 0.88) under identical analytical conditions. The number of concomitant medications was positively correlated with the incidence of serious adverse events, whereas the overall serious adverse event rate was higher for C-BUP than for L-BUP.

**Conclusion:**

L-BUP carries significant off-label use and formulation-specific medication error risks, with a positive correlation between concomitant medication use and serious adverse events. Many of these risks are preventable. These findings support targeted clinical and regulatory risk mitigation strategies for L-BUP, and provide evidence for improved post-marketing surveillance of complex modified-release formulations.

## 1 Introduction

According to statistics, the global volume of surgical procedures reached 312 million in 2012 and continues to grow (1). Effective perioperative pain management is central to improving postoperative patient outcomes. However, 30–80% of patients still experience moderate to severe postoperative pain, which significantly affects prognosis and patient satisfaction (2, 3). In recent years, healthcare regulatory agencies and academic organizations worldwide have issued perioperative pain management guidelines to promote the clinical application of multimodal analgesia, aiming to reduce opioid dependence and related adverse reactions (4, 5). As a core component of multimodal analgesia, local anesthetics achieve targeted analgesia through peripheral nerve blocks, wound infiltration, and other techniques, and have become a mainstream choice for postoperative pain management (6).

Bupivacaine is an amide-type long-acting local anesthetic that exerts its analgesic effect by blocking voltage-gated sodium channels in nerve fibers. The analgesic effect of a single injection of bupivacaine solution lasts only 4 to 6 h, which is insufficient to meet the demand for acute pain control within 72 h after surgery, and it is associated with a risk of rebound pain (7, 8). Liposomal bupivacaine (L-BUP), as a novel extended-release formulation, employs multivesicular liposome encapsulation technology to achieve slow drug release, providing analgesic effects for up to 72 h with a single dose and significantly reducing the frequency of postoperative analgesic administration, leading to its rapid expansion in clinical use after market approval (7). However, with its widespread clinical application, safety concerns have gradually emerged. Moreover, as a long-acting formulation, the continuous slow release of its active ingredient may also lead to more insidious delayed adverse reactions. The US FDA has issued safety alerts regarding the risk of cardiac adverse events associated with L-BUP. Existing studies have drawn inconsistent conclusions regarding the safety profile of L-BUP, and there is a lack of support from large-scale real-world data (9–11). Given the low incidence of serious adverse events such as local anesthetic systemic toxicity (LAST), prospective randomized controlled trials (RCTs) are limited by sample size and are therefore unable to systematically identify rare but serious risk signals. In contrast, the large-sample nature of spontaneous reporting databases helps to overcome this limitation (12).

Current safety studies on L-BUP have mostly focused on its pharmacological toxicity, with less attention paid to the increased risk resulting from deviations from recommended use in clinical practice. Compared with the inherent adverse drug reactions caused by its pharmacological toxicity, these instances of deviations from recommended use are risks that can be substantially reduced through measures such as medication education for clinicians, rational medication review systems, and standardized procedures (13). In recent years, several studies have suggested that factors such as insufficient clinical awareness of novel formulations and non-standard administration procedures, which fall under medication errors, may be important contributors to adverse events. However, no study has systematically analyzed the risk characteristics of medication errors associated with L-BUP (14, 15). Based on 2004–2025 data from the US FDA Adverse Event Reporting System (FAERS), this study used disproportionality analysis to systematically identify safety signals related to deviations from recommended use of L-BUP. Positive signals were screened at the Preferred Term (PT) level, and Standardized MedDRA Queries (SMQs) were used to focus on medication error risk. With conventional bupivacaine (C-BUP) as a comparator, we explored the formulation-specific characteristics of L-BUP-related medication errors and evaluated the association between polypharmacy and serious adverse events. This study provides real-world evidence for the safe and standardized use of L-BUP and a methodological reference for post-marketing surveillance and risk management of modified-release new drug formulations.

## 2 Methods

### 2.1 Dataset acquisition and processing

The data for this study were sourced from the FAERS. Reports from the first quarter of 2004 (Q1 2004) to the second quarter of 2025 (Q2 2025) were collected, covering the complete post-marketing period of both L-BUP and C-BUP. Using the unique CASEID as the linking variable, all reports listing bupivacaine as a primary suspect drug were extracted and merged. Duplicate reports were removed following the FDA-recommended algorithm: for the same CASEID, the report with the most recent FDA receipt date (FDA_DT) was retained; if both CASEID and FDA_DT were identical, the report with the largest PRIMARY _ID was retained. L-BUP records were identified by brand name (Exparel) and manufacturer (Pacira Pharmaceuticals, Inc.), while the remaining bupivacaine reports were classified as C-BUP cases.

### 2.2 Signal detection and data analysis

The study employed four complementary signal detection algorithms to ensure the robustness of the results: the Reporting Odds Ratio (ROR) method, the Proportional Reporting Ratio (PRR) method, the Bayesian Confidence Propagation Neural Network (BCPNN) method, and the Multi-item Gamma Poisson Shrinker (MGPS) method. All methods were based on 2 × 2 contingency tables, with relevant formulas and thresholds summarized in Table 1. A signal was defined as positive only when all four criteria were simultaneously met: (1) the lower limit of the 95% confidence interval (95% CI) of the ROR exceeded 1.0 and the number of reports ≥3; (2) the PRR was ≥2.0 with a chi-square (χ^2^) value ≥ 4 and the number of reports ≥3; (3) the lower limit of the 95% CI of the Information Component (IC − 2SD, IC_025_) from the BCPNN method exceeded 0; and (4) the lower limit of the 95% CI of the Empirical Bayesian Geometric Mean (EBGM_05_) from the MGPS method exceeded 1.0. If any one of these four conditions was not satisfied, the signal was classified as negative for that particular PT. Adverse event terminology was coded using PTs from the Medical Dictionary for Regulatory Activities (MedDRA version 28.0). A systematic analysis of medication error-related risks was conducted using the SMQ for “Medication error” (code: 20000224). All data processing and statistical analyses were performed using SAS software (version 9.4, SAS Institute, Cary, NC, USA).

**Table 1:** ROR, PRR, BCPNN, and EBGM calculation formulas and their thresholds.

Algorithms	Equation	Criteria
ROR	ROR = (a/b)/(c/d) = ad/bc	lower limit of 95% CI > 1, a ≥ 3
	95%CI = e^ln(ROR) ± 1.96(1/a + 1/b + 1/c + 1/d)^0.5^	
PRR	PRR = [a(c + d)]/[c(a + b)]	PRR ≥ 2, χ^2^≥4, a ≥ 3
	χ^2^=[(ad-bc)^2](a + b + c + d)/[(a + b)(c + d)(a + c)(b + d)]	
BCPNN	IC = log_2_[a(a + b + c + d)/(a + b)/(a + c)]	IC_025_ > 0
	95%CI = E(IC) ± 2 V(IC)^0.5	
MGPS	EBGM = a(a + b + c + d)/(a + c)/(a + b)	EBGM_05_ > 2
	95%CI = e^ln(EBGM) ± 1.96(1/a + 1/b + 1/c + 1/d)^0.5^	

a, number of reports containing both the target drug and target adverse drug reaction; b, number of reports containing other adverse drug reaction of the target drug; c, number of reports containing the target adverse drug reaction of other drugs; d, number of reports containing other drugs and other adverse drug reactions; 95%CI, 95% confidence interval; χ^2^, chi-squared; IC, information component; IC025, the lower limit of 95% CI of the IC; E(IC), the IC expectations; V(IC), the variance of IC; EBGM, empirical Bayesian geometric mean; EBGM05, the lower limit of 95% CI of EBGM.

### 2.3 Sensitivity analysis

To evaluate the formulation specificity of medication error signals for L-BUP, C-BUP was included as a comparator in the sensitivity analysis. Identical study timeframes, inclusion criteria for primary suspected drugs, MedDRA coding, and the four signal detection algorithms described in Section 2.2 were applied to C-BUP, so as to ensure methodological consistency and result comparability between the two groups. The analysis included: (1) Comparison of medication error SMQs signals: four signal detection algorithms were separately calculated for L-BUP and C-BUP under the SMQ term “Medication error” (SMQ code: 20000224), followed by direct intergroup comparison; (2) Comparison of serious adverse event outcomes: based on patient adverse event outcomes, the proportion of serious adverse event reports between L-BUP and C-BUP was compared using both absolute case counts and constituent ratios. Serious adverse events were defined as those resulting in the following seven outcomes: Life-Threatening (LT), Initial or Prolonged Hospitalization (HO), Disability (DS), Death (DE), Congenital Anomaly (CA), Intervention Required to Prevent Permanent Impairment or Damage (RI), and Other Adverse Events (OT).

This parallel analysis serves as a comparative reference to contextualize whether elevated medication error signals are formulation-specific to L-BUP or reflect broader reporting patterns within the bupivacaine class. C-BUP was used as a comparator rather than a strict negative control because the two formulations differ in clinical indications, dosing regimens, and target populations; therefore, any observed differences should be interpreted with consideration of potential confounding by practice patterns, reporting behaviors, and population characteristics.

## 3 Results

### 3.1 Descriptive analysis

**Figure 1:**
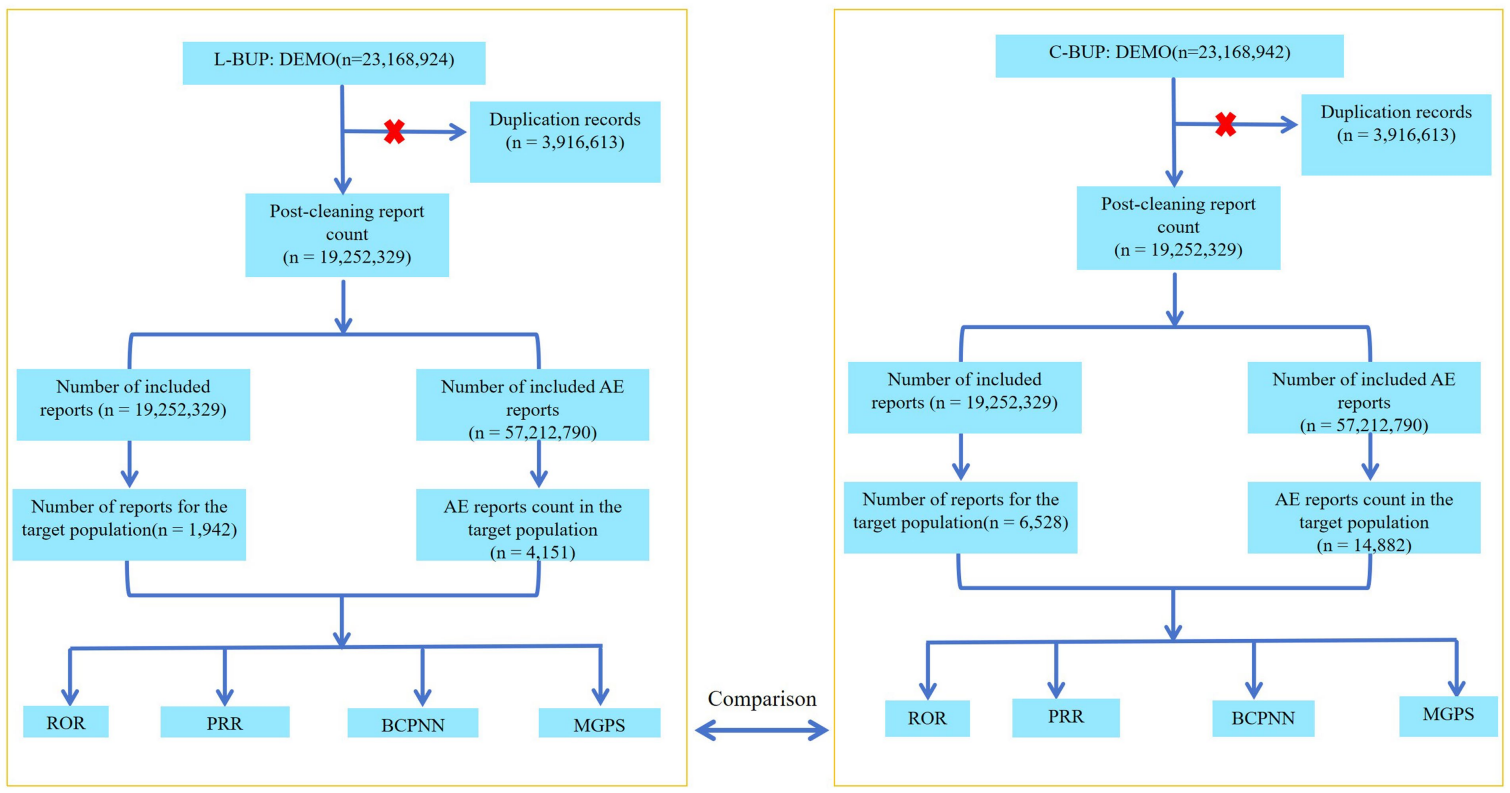
Flow chart of data extraction for FAERS database analysis.

From Q1 2004 to Q2 2025, a total of 19,252,329 safety reports were extracted from the FAERS database for analysis. Among these, 4,151 reports listed L-BUP as the primary suspect drug, corresponding to 1,942 patients; meanwhile, 14,882 reports listed C-BUP as the primary suspect drug, involving 6,528 patients. The data processing workflow of the FAERS database is illustrated in Figure 1. For L-BUP reports, the median patient age was 57 years, with females accounting for 31.9% and males 16.2%. For C-BUP reports, the median patient age was 48 years, with females accounting for 46.8% and males 22.2%. Reports of both bupivacaine formulations were predominantly from North America, accounting for 97.2% of L-BUP reports and 72.2% of C-BUP reports. Most reports were submitted by healthcare professionals, accounting for 77.0% of L-BUP reports and 65.6% of C-BUP reports, indicating a relatively high overall data quality. Detailed data are presented in Table 2.

**Table 2:** Characteristics of adverse events among patients using liposomal bupivacaine and conventional bupivacaine based on the FAERS database.

Characteristics	Liposomal bupivacaine	Conventional bupivacaine
Sex, *n* (%)		
Female	619 (31.9%)	3,056 (46.8%)
Male	314 (16.2%)	1,452 (22.2%)
Not specified	1,009 (51.9%)	2,020 (31.0%)
Age (years), *n* (%)		
<18	29 (1.5%)	191 (2.9%)
18–44	132 (6.8%)	1,437 (22.0%)
45–64	232 (11.9%)	931 (14.3%)
≥65	189 (9.7%)	934 (14.3%)
Not specified	1,360 (70.1%)	3,035 (46.5%)
Median (Q1, Q3)	57 (42–67)	48 (31–66)
Reporters, *n* (%)		
Consumer	425 (21.9%)	1,523 (23.3%)
Lawyer	1 (0.1%)	292 (4.5%)
Not specified	21 (1.0%)	429 (6.6%)
Other health-professional	74 (3.8%)	1,189 (18.2%)
Pharmacist	421 (21.7%)	1,832 (28.1%)
Physician	1,000 (51.5%)	1,263 (19.3%)
Reported continents, *n* (%)		
North America	1,889 (97.2%)	4,715 (72.2%)
Europe	21 (1.0%)	937 (14.4%)
Asia	7 (0.5%)	522 (8.0%)
Not specified	25 (1.3%)	204 (3.1%)
Others	0 (0)	150 (2.3%)

### 3.2 Analysis of L-BUP-related signals in the FAERS database at the PT level

In this study, four commonly used signal detection methods were employed to identify adverse event signals associated with L-BUP from the FAERS database. Based on the frequency of positive signals, the top 20 positive PTs were selected. As shown in Table 3, among the top 20 positive signals by frequency, 7 PTs were related to deviations from recommended use, accounting for 35% of the top 20 positive signals. According to the reported frequency, the top PTs related to deviations from recommended use were as follows: “Off-label use” (*n* = 238), “Labeled drug–drug interaction medication error” (*n* = 191), “Extra dose administered” (*n* = 52), “Product administered to patient of inappropriate age” (*n* = 46), “Inappropriate schedule of product administration” (*n* = 45), “Prescribed overdose” (*n* = 34), and “Product administration error” (*n* = 27). All these PTs generated signals exceeding their respective thresholds across the four disproportionality analysis algorithms. Notably, “Off-label use” had the highest number of reports among all positive signals, with a ROR of 4.5 (95% CI: 3.9–5.1), PRR of 4.3 (χ^2^ = 604.5), IC of 2.1 (IC₀₂₅ = 1.9), and EBGM of 4.3 (EBGM₀₅ = 3.8). “Labeled drug–drug interaction medication error” exhibited the strongest signal strength, with an ROR of 332.7 (95% CI: 287.3–385.3), PRR of 317.4 (χ^2^ = 58897.7), IC of 8.3 (IC₀₂₅ = 6.7), and EBGM of 310.3 (EBGM₀₅ = 267.9). These signals suggest that non-standard clinical use of L-BUP may be associated with an increased risk of adverse events, and further clinical studies are needed to validate the causal relationship. This issue requires urgent attention from both clinical practitioners and regulatory authorities.

**Table 3:** Top 20 preferred terms ranked by frequency of positive signals for liposomal bupivacaine.

PT	Case	ROR (95% CI)	PRR (χ^2^)	IC (IC_025_)	EBGM (EBGM_05_)
Off label use	238	4.5 (3.9–5.1)	4.3 (604.5)	2.1 (1.9)	4.3 (3.8)
Labeled drug–drug interaction medication error	191	332.7 (287.3–385.3)	317.4 (58897.7)	8.3 (6.7)	310.3 (267.9)
No adverse event	182	15.9 (13.8–18.5)	15.3 (2435.9)	3.9 (3.6)	15.3 (13.2)
Hospitalization	125	13.0 (10.9–15.5)	12.6 (1341.6)	3.7 (3.3)	12.6 (10.6)
Pain	124	3.0 (2.6–3.6)	2.9 (165.3)	1.6 (1.3)	2.9 (2.5)
Hypoaesthesia	104	10.5 (8.6–12.7)	10.2 (865.6)	3.4 (2.9)	10.2 (8.4)
Extra dose administered	52	21.8 (16.5–28.6)	21.5 (1013.6)	4.4 (3.6)	21.4 (16.3)
Procedural pain	49	27.4 (20.6–36.3)	27.1 (1228.3)	4.8 (3.7)	27.0 (20.4)
Urinary retention	49	22.2 (16.8–29.5)	22.0 (980.1)	4.5 (3.5)	21.9 (16.6)
Product administered to patient of inappropriate age	46	52.1 (39.0–69.8)	51.6 (2273.4)	5.7 (4.2)	51.4 (38.4)
Inappropriate schedule of product administration	45	2.8 (2.1–3.8)	2.8 (52.3)	1.5 (1.0)	2.8 (2.1)
Hypotension	43	3.2 (2.4–4.4)	3.2 (65.2)	1.7 (1.2)	3.2 (2.4)
Seizure	37	3.2 (2.3–4.4)	3.2 (55.4)	1.7 (1.1)	3.2 (2.3)
Prescribed overdose	34	27.1 (19.4–38.1)	26.9 (847.4)	4.8 (3.5)	26.9 (19.2)
Paraesthesia	32	3.0 (2.1–4.3)	3.0 (42.5)	1.6 (1.0)	3.0 (2.1)
Infection	31	3.3 (2.3–4.7)	3.3 (49.2)	1.7 (1.1)	3.3 (2.3)
Postoperative wound infection	29	56.7 (39.3–81.8)	56.3 (1569.2)	5.8 (3.8)	56.1 (38.9)
Therapeutic response shortened	28	15.3 (10.6–22.2)	15.2 (372.0)	3.9 (2.8)	15.2 (10.5)
Local anesthetic systemic toxicity	27	564.9 (384.0–831.1)	561.3 (14509.2)	9.1 (4.2)	539.3 (366.6)
Product administration error	27	7.8 (5.3–11.4)	7.7 (158.4)	3.0 (2.1)	7.7 (5.3)

### 3.3 Safety signal detection results related to medication error at the SMQ level

On the basis of multiple positive signals related to deviations from recommended use identified at the PT level, we further focused on the more specific “medication errors” and conducted a systematic signal detection using the SMQ for medication errors. The results showed a total of 457 medication error-related reports in the L-BUP target population, which were consistently detected as a strong positive signal by all algorithms: ROR = 4.2 (95% CI: 3.8–4.6), PRR = 3.8 (χ^2^ = 980.0), IC = 1.9 (IC₀₂₅ = 1.8), and EBGM = 3.8 (EBGM₀₅ = 3.5). This suggests that the liposomal formulation may carry a specific risk of medication errors. We further analyzed the PTs under the SMQ for medication errors. A total of 25 PTs were detected under this SMQ. Disproportionality signals were computed using the same four algorithms, and based on the combined criterion of the four methods, 7 PTs were identified as positive signals. Detailed results are presented in Table 4. In descending order of report frequency, these were:

**Table 4:** Positive preferred terms signals under the medication error for liposomal bupivacaine.

PT	Case	ROR (95% CI)	PRR (χ^2^)	IC (IC_025_)	EBGM (EBGM_05_)
Labeled drug–drug interaction medication error	191	332.7 (287.3–385.3)	317.4 (58897.7)	8.3 (6.7)	310.3 (267.9)
Extra dose administered	52	21.7 (16.5–28.6)	21.5 (1013.6)	4.4 (3.5)	21.4 (16.3)
Product administered to patient of inappropriate age	46	52.1 (38.9–69.8)	51.6 (2273.3)	5.7 (4.2)	51.4 (38.4)
Inappropriate schedule of product administration	45	2.8 (2.1–3.8)	2.8 (52.3)	1.5 (1.0)	2.8 (2.1)
Product administration error	27	7.8 (5.3–11.4)	7.7 (158.4)	2.9 (2.1)	7.7 (5.3)
Incorrect route of product administration	16	4.4 (2.7–7.2)	4.4 (42.0)	2.1 (1.2)	4.4 (2.7)
Accidental overdose	11	4.5 (2.5–8.1)	4.5 (29.9)	2.2 (0.9)	4.5 (2.5)

“Labeled drug-drug interaction medication error” (*n* = 191), with ROR = 332.7 (95% CI: 287.3–385.3), PRR = 317.4 (χ^2^ = 58897.7), IC = 8.3 (IC₀₂₅ = 6.7), EBGM = 310.3 (EBGM₀₅ = 267.9);

“Extra dose administered” (*n* = 52), with ROR = 21.7 (95% CI: 16.5–28.6), PRR = 21.5 (χ^2^ = 1013.6), IC = 4.4 (IC₀₂₅ = 3.5), EBGM = 21.4 (EBGM₀₅ = 16.3);

“Product administered to patient of inappropriate age” (*n* = 46), with ROR = 52.1 (95% CI: 38.9–69.8), PRR = 51.6 (χ^2^ = 2273.3), IC = 5.7 (IC₀₂₅ = 4.2), EBGM = 51.4 (EBGM₀₅ = 38.4);

“Inappropriate schedule of product administration” (*n* = 45), with ROR = 2.8 (95% CI: 2.1–3.8), PRR = 2.8 (χ^2^ = 52.3), IC = 1.5 (IC₀₂₅ = 1.0), EBGM = 2.8 (EBGM₀₅ = 2.1);

“Product administration error” (*n* = 27), with ROR = 7.8 (95% CI: 5.3–11.4), PRR = 7.7 (χ^2^ = 158.4), IC = 2.9 (IC₀₂₅ = 2.1), EBGM = 7.7 (EBGM₀₅ = 5.3);

“Incorrect route of product administration” (*n* = 16), with ROR = 4.4 (95% CI: 2.7–7.2), PRR = 4.4 (χ^2^ = 42.0), IC = 2.1 (IC₀₂₅ = 1.2), EBGM = 4.4 (EBGM₀₅ = 2.7);

“Accidental overdose” (*n* = 11), with ROR = 4.5 (95% CI: 2.5–8.1), PRR = 4.5 (χ^2^ = 29.9), IC = 2.2 (IC₀₂₅ = 0.9), EBGM = 4.5 (EBGM₀₅ = 2.5).

The above results indicate that the risk of medication errors for the target drug is significantly higher than that for other drugs. The risks are primarily concentrated in key areas such as management of drug interactions, appropriate patient selection, regimen design, and standardization of administration procedures.

### 3.4 Risk analysis of concomitant medication with L-BUP

**Figure 2:**
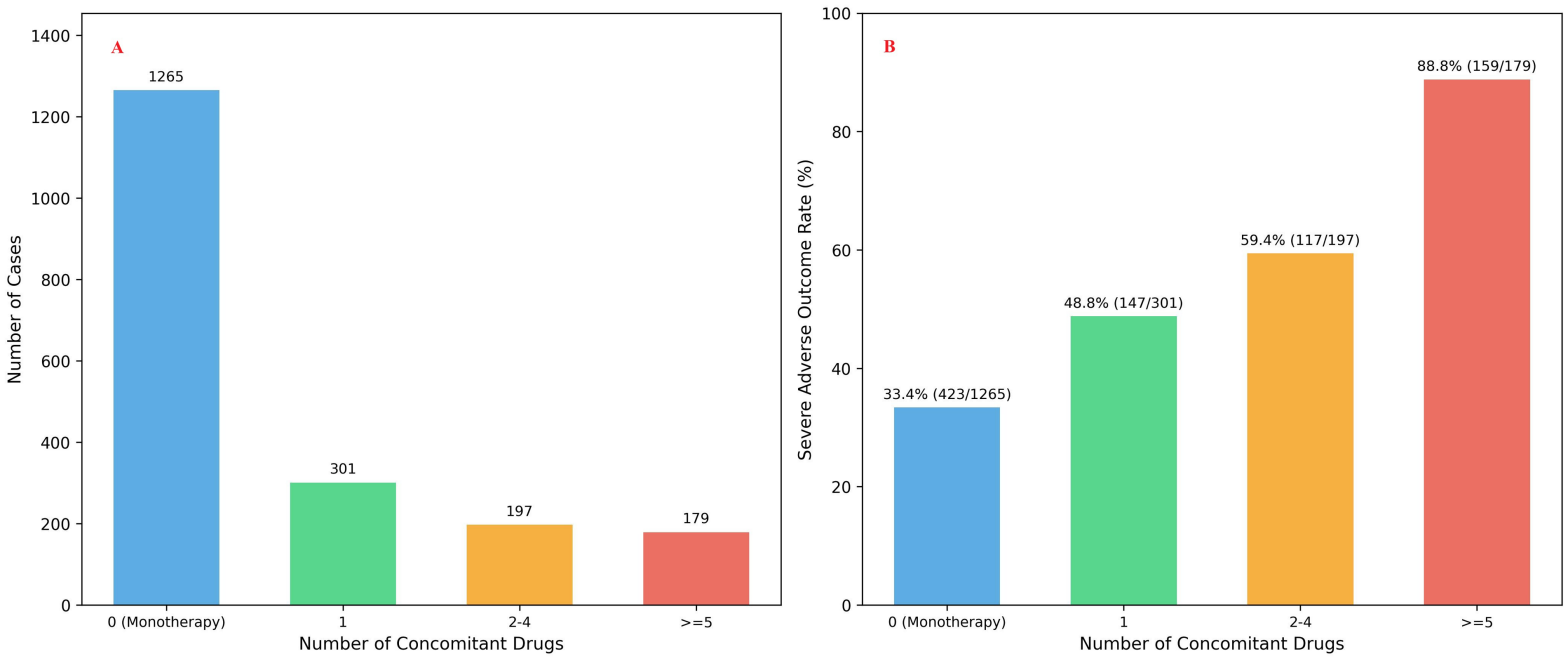
Concomitant medications in adverse event reports of liposomal bupivacaine. **(A)** Number of reports with concomitant medications; **(B)** number of serious adverse events and concomitant medications.

Based on the above risk analysis results for L-BUP, the “Labeled drug-drug interaction medication error” was identified as a risk signal, ranking second in the number of reports among all positive signal PTs and first among the positive PTs within the Medication error SMQ. Therefore, this study further analyzed the concomitant medication risk associated with L-BUP. The data showed that among the 1,942 patients with adverse events, 1,265 (65.1%) received L-BUP alone, 301 (15.5%) were co-administered with 1 concomitant medication, 197 (10.1%) with 2–4 concomitant medications, and 179 (9.2%) with ≥5 concomitant medications. The incidence rates of serious adverse events under different concomitant medication regimens were as follows: 33.4% (423/1,265) in the L-BUP monotherapy group, 48.8% (147/301) in patients with 1 concomitant drug, 59.4% (117/197) in those with 2–4 concomitant drugs, and 88.8% (159/179) in those with ≥5 concomitant drugs. The detailed results are shown in Figure 2.

**Figure 3:**
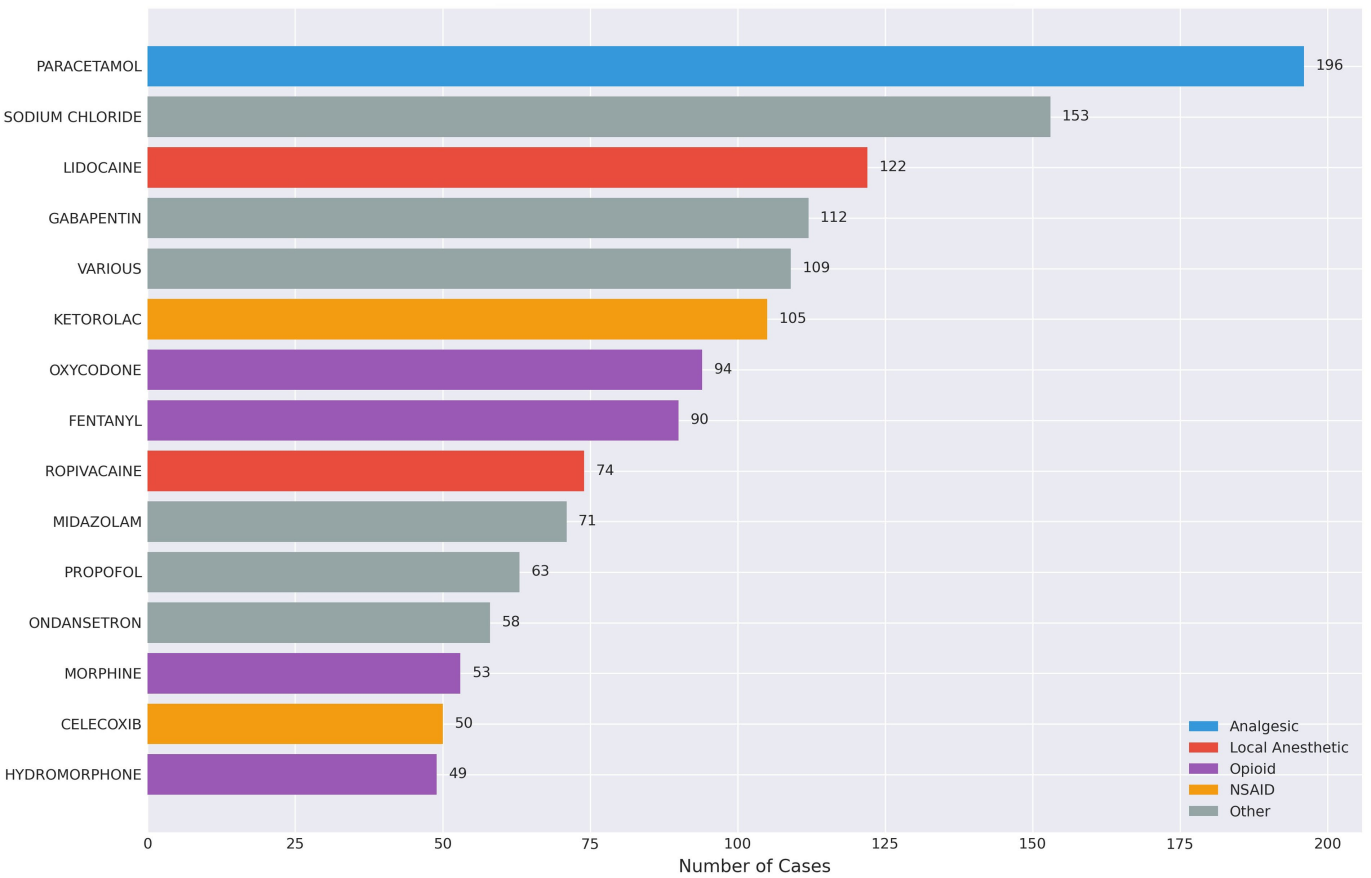
Top 15 concomitant drugs in adverse event reports of liposomal bupivacaine.

Analysis of specific concomitant medications revealed that acetaminophen was the most frequently co-administered drug, accounting for 196 cases (10.1%), followed by sodium chloride, lidocaine, gabapentin, and ketorolac. The main classes involved were non-steroidal anti-inflammatory drugs, local anesthetics, and opioid analgesics (Figure 3). Co-administration of local anesthetics may increase the risk of LAST, while co-administration of opioids may synergistically enhance the potential risk of respiratory depression.

### 3.5 Sensitivity analysis

**Figure 4:**
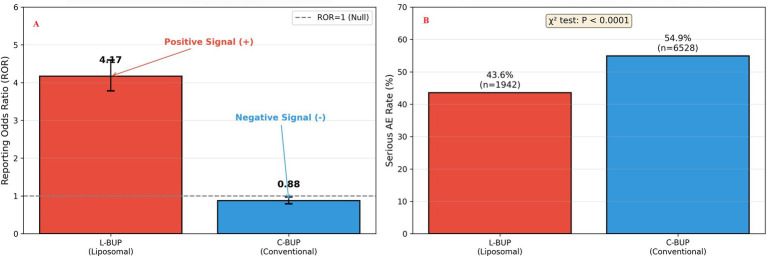
Comparison of adverse events between liposomal bupivacaine and conventional bupivacaine. **(A)** Comparison of “Medication error” (SMQ) signals between the two formulations. **(B)** Comparison of serious adverse events between the two formulations.

To verify the specificity of the medication error signal for L-BUP, we performed a sensitivity analysis with C-BUP as the comparator. The analysis compared the two formulations in terms of SMQs-level signal characteristics and patient-level outcomes. A direct comparison of the “Medication error (SMQ)” signals between L-BUP and C-BUP showed significant differences (Figure 4A). L-BUP exhibited a positive signal (ROR = 4.17, 95% CI: 3.78–4.60; all four criteria were satisfied), whereas C-BUP showed a negative signal (ROR = 0.88, 95% CI: 0.79–0.97; all four criteria were negative). The ROR (L-BUP/C-BUP) was 4.75. The significant difference in signal direction (positive vs. negative) confirms that the medication error signal detected for L-BUP is formulation-specific rather than a class effect of bupivacaine. Patient-level outcome analysis revealed an unexpected finding: the incidence of serious AE was significantly higher for C-BUP (54.9%, 3,586/6,528) than for L-BUP (43.6%, 846/1,942) (χ^2^ = 77.1, *p* < 0.0001) (Figure 4B).

Collectively, these comparative results confirm that the detected signals are not attributable to surveillance bias or reporting artifacts, but rather represent safety issues specific to the liposomal formulation.

## 4 Discussion

Constrained by strict inclusion criteria and standardized dosing, clinical trials rarely capture adverse events related to deviations from recommended use (16). This real-world pharmacovigilance study is the first to systematically reveal formulation-specific deviations from recommended use associated with L-BUP. This finding moves beyond the conventional focus on adverse reactions caused solely by pharmacological and toxicological effects, and provides a new analytical framework for the post-marketing lifecycle risk management of improved new drugs.

### 4.1 Reasons for deviations from recommended use of L-BUP

The safety signals for L-BUP observed in this study, predominantly off-label use and medication errors, may be attributed to the unique physicochemical properties of the liposomal formulation together with regulatory and practical factors.

First, the liposomal design of L-BUP exhibits certain physicochemical incompatibilities with other drugs (17), whereas C-BUP is frequently mixed with other amide local anesthetics in clinical practice (18). Contact with non-encapsulated bupivacaine, lidocaine, or other local anesthetics disrupts the liposomal membrane, triggering immediate drug release and altering the pharmacokinetic profile (17). This may negate its extended-release advantage and increase the risk of drug-related adverse events, including the risk of LAST (19). Accordingly, the L-BUP label explicitly advises against admixing. The extreme signal strength for “Labeled drug-drug interaction medication error” (ROR = 332.7) suggests that this incompatibility is not always observed in practice, likely reflecting habits carried over from long-established C-BUP use. Second, L-BUP has relatively narrow approved indications, mainly surgical wound infiltration and selected peripheral nerve blocks, with age restrictions and single-administration dosing. Clinical demand for prolonged postoperative analgesia, however, frequently extends to off-label scenarios such as intra-articular administration (20). This gap between the limited label and broader analgesic needs likely contributes to off-label use, which was the most frequently reported deviation in our analysis (21, 22). The additional medication error signals detected, including inappropriate dosing, scheduling, route, and patient selection, further indicate that off-label practices often proceed without adequate standardization. Third, L-BUP handling requirements differ substantially from those of C-BUP, including restrictions on diluent type, needle gauge, filtration, and pre-administration conditions. These constraints demand new knowledge and skills, yet system-level safeguards have lagged behind clinical adoption, which may partly account for the “product administration error” signal (9, 23).

Collectively, these factors form a mechanistic triad of physicochemical incompatibility, demand-driven off-label use, and increased procedural complexity. This triad may explain the spectrum of deviation signals observed, from inappropriate patient selection to dosing and administration errors. Similar challenges have been reported for liposomal amphotericin B, reinforcing the view that complex sustained-release injectable formulations require greater professional awareness, enhanced training, and more rigorous regulatory oversight (24).

### 4.2 Effect of polypharmacy on the safety of L-BUP

A crude association was observed between a greater number of concomitant medications and a higher proportion of serious adverse event reports among L-BUP users. This finding requires cautious interpretation. In spontaneous reporting databases, a higher number of co-reported drugs often reflects greater patient complexity—more severe disease, advanced age, more comorbidities, and longer hospital stays. Each of these factors independently raises the risk of serious adverse events (25, 26).

Nonetheless, the concomitant medication analysis yields several informative observations. First, the proportion of serious adverse events was lower for L-BUP than for C-BUP. This difference may partly reflect the narrower approved indications of L-BUP, which is restricted to selected peripheral nerve blocks and surgical wound infiltration and is not approved for neuraxial administration. This limitation likely reduces the risk of severe events from inadvertent intravascular injection, a recognized hazard of C-BUP used epidurally or spinally (27, 28). Second, among the top 10 most frequently co-administered drugs, lidocaine (third) and ropivacaine (ninth) are both amide local anesthetics. Their presence directly reinforces the “Labeled drug-drug interaction medication error” signal, suggesting that the interactions captured by this term predominantly involve co-administration with other amide local anesthetics. That two different amide agents appear among the most common concomitant drugs supports the concern that mixing L-BUP with other local anesthetics occurs with appreciable frequency (9).

### 4.3 Strategies for preventing medication errors with L-BUP

The spectrum of deviation signals identified in this study highlights the need for a full-chain risk management strategy covering prescribing, administration, and postoperative monitoring. In the prescribing decision-making, it is recommended to embed a mandatory route of administration verification function into the electronic medical record system to automatically block prescriptions involving contraindicated routes such as intravenous injection and intra-articular injection. Given the markedly strong signal of “Labeled drug-drug interaction medication errors”, pop-up warnings should be implemented for concurrent prescription of other amide local anesthetics. In administration procedures, standardized operating procedures should be established, including strict adherence to technical specifications such as pre-injection aspiration confirmation and slow fractional injection. A dual-verification mechanism for the route of administration should be implemented. Additionally, a credentialing system for healthcare professionals should be established to ensure that operators possess the competence to identify contraindicated routes and manage LAST. In the postoperative monitoring phase, given that the sustained-release properties of L-BUP may lead to delayed adverse reactions, the monitoring window should be extended to 72 h, with a stratified monitoring approach: routine monitoring for low-risk patients, and extended hospitalization or intensive care unit (ICU) care for intermediate-to-high-risk patients. These measures can be integrated within existing institutional frameworks and address the gap between the procedural complexity of this liposomal formulation and the safeguards currently in place.

### 4.4 Limitations

This study has several limitations inherent to the FAERS database. First, as a spontaneous reporting system, FAERS is subject to reporting bias (29). The quality of reports is variable; severe, rare, or widely publicized adverse events are more likely to be reported, whereas mild or routine events tend to be underreported. Consequently, the data may not fully reflect the comprehensive safety profile of L-BUP. Second, information bias is present due to incomplete clinical data (30). The database lacks detailed information on patients’ underlying diseases, disease severity, and concomitant medications, making it difficult to distinguish adverse events directly attributable to the drug from those caused by underlying comorbidities, disease progression, or polypharmacy interactions. Third, FAERS data can only establish statistical associations between drugs and adverse events, and cannot directly demonstrate causality (31). Thus, causal inference is subject to inherent bias.

To mitigate these biases, we implemented several methodological safeguards. Consistent disproportionality signals were detected across multiple analytical approaches, including PT-level and SMQ-level analyses using four complementary signal detection algorithms. Sensitivity analysis with C-BUP as a comparator confirmed the formulation specificity of the observed signals. Furthermore, the clinical plausibility of our findings is supported by physicochemical properties, labeling restrictions, and procedural factors discussed above.

Future prospective pharmacoepidemiologic studies, such as large-scale observational cohorts or registry-based studies, are warranted to validate the causal relationship between L-BUP and the identified deviations from recommended use, and to quantify the absolute risk in real-world clinical practice.

## 5 Conclusion

This real-world pharmacovigilance study demonstrates that adverse event signals associated with L-BUP cluster prominently around deviations from recommended use, particularly medication errors involving drug–drug interactions and off-label administration. The signal specificity was confirmed by the absence of comparable medication error signals for C-BUP and by the consistency of findings across multiple detection algorithms and analytical levels. These deviations appear largely attributable to a combination of physicochemical incompatibilities, a gap between limited labeled indications and broad analgesic demand, and increased procedural complexity inherent to the liposomal formulation. Importantly, many of the identified risks are preventable. Targeted safeguards across the prescribing, administration, and monitoring chain could substantially mitigate harm without restricting access to this effective analgesic. This study suggests that post-marketing safety surveillance of complex modified-release formulations should systematically attend to deviations from recommended use, proactively evaluate their signal characteristics and underlying causes, and implement targeted measures to reduce potentially preventable adverse events.

## Data Availability

The original contributions presented in the study are included in the article/supplementary material, further inquiries can be directed to the corresponding authors.
